# A Temperature Compensation Method for aSix-Axis Force/Torque Sensor Utilizing Ensemble hWOA-LSSVM Based on Improved Trimmed Bagging

**DOI:** 10.3390/s22134809

**Published:** 2022-06-25

**Authors:** Xuhao Li, Lifu Gao, Huibin Cao, Yuxiang Sun, Man Jiang, Yue Zhang

**Affiliations:** 1 Institutes of Physical Science and Information Technology, Anhui University, Hefei 230093, China; dawy@mail.ustc.edu.cn (X.L.); zy422@mail.ustc.edu.cn (Y.Z.); 2 Hefei Institutes of Physical Science, Chinese Academy of Sciences, Hefei 230031, China; lifugao@iim.ac.cn (L.G.); yxsun@iim.ac.cn (Y.S.); mjiang@iim.ac.cn (M.J.); 3 School of Science Island, University of Science and Technology of China, Hefei 230026, China

**Keywords:** temperature compensation, six-axis force/torque sensor, whale optimization algorithm, bagging, least square support vector machine

## Abstract

The performance of a six-axis force/torque sensor (F/T sensor) severely decreased when working in an extreme environment due to its sensitivity to ambient temperature. This paper puts forward an ensemble temperature compensation method based on the whale optimization algorithm (WOA) tuning the least-square support vector machine (LSSVM) and trimmed bagging. To be specific, the stimulated annealing algorithm (SA) was hybridized to the WOA to solve the local entrapment problem, and an adaptive trimming strategy is proposed to obtain the optimal trim portion for the trimmed bagging. In addition, inverse quote error (invQE) and cross-validation are employed to estimate the fitness better in training process. The maximum absolute measurement error caused by temperature decreased from 3.34% to $3.9\times 10^{-3}\%$ of full scale after being compensated by the proposed method. The analyses of experiments illustrate the ensemble hWOA-LSSVM based on improved trimmed bagging improves the precision and stability of F/T sensors and possesses the strengths of local search ability and better adaptability.

## 1. Introduction

Force/torque sensors are widely applied in manipulators to obtain feedback on force and torque, which can help researchers achieve close-loop control. When working in extreme conditions, for instance, in space [1] or in deep-sea [2,3] applications, F/T sensors will suffer severe characteristic drift due to the wide range of ambient temperature. The characteristic drifts will cause bias between measured values and real values, consequently decreasing the precision of measurements. Thus, the effects of temperature should be compensated for to alleviate and even eliminate the characteristic drifts of F/T sensors.

The impacts of temperature on strain gauge sensors are omnifarious. The thermal effects of strain gauges and the different dilatation coefficients between strain gauges and elastic bodies are two major factors which cause characteristic drifts. Hardware and software methods are two ways to compensate for the impacts of temperature. Compensating methods focusing on hardware aim to eliminate the temperature’s impact on the regulating circuit [4,5,6]. However, compensating on the circuit is usually costly and lacks flexibility. More importantly, it cannot meet the requirement of precision. Thus, hardware compensating methods are used as a kind of auxiliary means in practice [7]. Software compensating methods aim to build inverse models between ambient temperature and thermal output. Sensors compensate for the characteristic drift according to the thermal output obtained by inverse models. Software compensation methods are powerful, flexible, and easily applied to various sensors. In view of these advantages, scholars proposed lots of software compensating methods, such as least square method (LSM), support vector machine (SVM) [8,9], and all kinds of artificial neutral networks (ANN) [10,11,12]. LSM is convenient and intuitive, so researchers commonly apply it to solve linear and low rank non-linear fitting problems. However, the compensating performances of LSM are not as good as expected because rank non-linear error is usually involved in characteristic drifts. In practice, LSM is still applied to temperature compensation due to its simplicity when the calculating capacity is limited by the process unit size. The applications of ANN have been more and more extensive in recent years due to their prominent nonlinear fitting ability, and scholars proposed massive compensation methods based on ANN [13,14,15]. However, compensating methods based on ANN suffer from two issues: low convergence rate and complex network structures. SVM derives from statistical learning theory and structural risk minimization (SRM). Convex optimization algorithms, such as quadratic programs, are adopted to solve SVM. Suykens J.A.K. and Vandewalle J. reformulated standard SVM and proposed the least-square support vector machine (LSSVM) [16]. LSSVM inherits remarkable abilities for solving nonlinear and high rank regression problems from SVM and provides some improvements, including adopting a binary norm in the objective function and converting the optimization problem to solve the linear Karush–Kuhn–Tucker (KKT) condition. Zhang et al. [17] proposed a LSSVM error compensation model to accurately estimate the state of health (SOH) of lithium-ion batteries. Tan et al. [18] predicted the thermal error of machine tool spindles using the segment fusion least-square support vector machine (SF-LSSVM) and improved the prediction precision by up to 51%.

Though LSSVM is powerful in nonlinear fitting, its performance is significantly influenced by the choice of its parameters. Obtaining parameters of LSSVM by blind search is a costly task; therefore, various optimization algorithms are adopted by scholars, such as the Cuckoo search algorithm (CSA), particle swarm optimization (PSO), and the sparrow search algorithm (SSA) [19,20,21]. The whale optimization algorithm (WOA) is a meta-heuristic optimization algorithm which is inspired by the bubble-net hunting behavior of humpback whales [22]. The WOA obtains competitive performances in global exploration, has high flexibility compared with other optimization algorithms, and owns a simple structure and few parameters, which make it easy to be adopted in many fields. Although the WOA can efficiently locate the latent positions of the solution in the exploration phase, it suffers from a low convergence rate in the exploitation phase and has a tendency for being trapped in local optima [23,24]. Several measures are taken to offset these shortcomings of WOA, and embedding a high-efficiency local algorithm into WOA is gaining widespread traction. Tong [25] designed a hybrid algorithm framework for WOA, and verified the framework by embedding differential evolution (DE) and the backtracking search optimization algorithm (BSA) into WOA. Nadim et al. [26] hybridized WOA and DE to solve multi-objective virtual machine scheduling in cloud computing. Based on the aforesaid idea, this paper enhances the exploitative ability of the WOA by adopting the stimulated annealing algorithm (SA).

Ensemble method is a series of machine learning methods; boosting and bagging are representatives. They follow the "sampling–training–combining" workflow [27]. Ensemble methods are in the spotlight of the machine learning field for their remarkable improvements in accuracy compared to single learners. Bagging adopts bootstrap sampling to obtain training data and combines the output of each learner by the most common strategy, such as voting for classification and averaging for regression [28]. Bagging can reduce the variance of base learners, especially when those base learners are unstable. In view of improving the performance of any classifier, Joossens et al. proposed trimmed bagging, which only averages outputs from the best base learners, rather than all of them, when applying the bagging method. LSSVM is a stable learning method, and the turbulence of training set can have a subtle impact on it. The standard bagging algorithm works not well, and LSSVM acts as the base learner; therefore, trimmed bagging is adopted in the proposed method.

In this study, an ensemble temperature compensation approach is proposed which adopts LSSVM as the base learner and trimmed bagging as the ensemble framework. To achieve the optimal performance of LSSVM, the hybrid whale optimization algorithm (hWOA) was used to configure parameters in LSSVM, which introduced the simulated annealing algorithm (SA) to WOA. In addition, inverse quoted error (inv QE) was taken to weight aggregate outputs of base learners for its extensive usage in practice. Finally, a temperature compensation model was established by ensemble hWOA-LSSVM based on trimmed bagging.

## 2. Basic Algorithm

### 2.1. Least-Square Support Vector Machine (LSSVM)

The LSSVM is used vastly in many fields, such as classification, pattern recognition, and regression. The replacement of non-equality constraints by equality constrains in formulation is the main distinction between LSSVM and standard SVM.

Given a training set $D_{t}={\left\{x_{i},y_{i}\right\}}_{i=1}^{N}$, where $x_{i}\in R^{n}$ is the input vector and $y_{i}\in R$ is the output vector, the LSSVM can be described as:

(1) $$f\left(x\right)=w^{T}\varphi \left(w\right)+b$$

where

$w=(w_{1};w_{2};\cdots ;w_{d})$ is a normal vector of the hyperplane,
*b* is a bias that decides the distance between the hyperplane and origin point,
φ(·) is a mapping function which maps input data into a higher dimension space.

The aim of a regression task is to minimize the difference between predicted output $f(x_{i})$ and real output $y_{i}$. Hence, according to the theory of regulation, the optimization object of LSSVM can be written as

(2) $$\begin{array}{c} \min_{w,b,e}\;\;\frac{1}{2}w^{T}w+\frac{1}{2}\gamma \sum_{i=1}^{N}e_{i}^{2},\\ s.t.\;f\left(x_{i}\right)-y_{i}=e_{i},i=1,2,\cdots ,N\;. \end{array}$$

where γ is the regulation item which controls the balance between the flexibility and accuracy of LSSVM; $e_{i}$ denotes the error between the predicted output and real output.

We can define the Lagrange function as

(3) $$\begin{array}{cc} L(w,b,e;a)= & \frac{1}{2}w^{T}w+\frac{1}{2}\gamma \sum_{i=1}^{N}e_{i}^{2}\\ \; & -\sum_{i=1}^{N}a_{i}\left[f\left(x_{i}\right)-y_{i}-e_{i}\right]\;, \end{array}$$

where $a_{i}\;\left(i=1,2,\cdots ,N\right)$ are Lagrange coefficients and L(w,b,e;a) should meet the Karush–Kuhn–Tucker (KKT) condition, which is a necessary condition to obtain optimal solution in nonlinear programming [29].

Substitute Equation (1) into Equation (3), and then the KKT condition can be described as follows:

(4) $$\begin{array}{cc} \frac{\partial L}{\partial w} & =0\to w=\sum_{i=1}^{N}a_{i}\varphi \left(x_{i}\right),\\ \frac{\partial L}{\partial b} & =0\to \sum_{i=1}^{N}a_{i}=0,\\ \frac{\partial L}{\partial e_{i}} & =0\to a_{i}=\gamma e_{i},\\ \frac{\partial L}{\partial a_{i}} & =0\to w^{T}\varphi \left(x_{i}\right)+b+e_{i}-y_{i}=0. \end{array}$$

After eliminating γ and w, Equation (4) can be rewritten immediately as the following set of linear equations:

(5) $$\left[\begin{array}{cc} 0 & \Theta \\ \Theta & \Omega +\gamma^{-1}I \end{array}\right]\left[\begin{array}{c} b\\ a \end{array}\right]=\left[\begin{array}{c} 0\\ Y \end{array}\right]\;,$$

where

*I* is the identity matrix,
Θ=[1,1,⋯,1],
$Y=[y_{1},y_{2},\cdots ,y_{N}]\;$.

Considering the complexity of calculating the inner product in high dimensionality, we apply kernel trick here—namely,

(6) $$\begin{array}{c} \Omega_{ij}=\varphi {\left(x_{i}\right)}^{T}\varphi \left(x_{j}\right)=\kappa \left(x_{i},x_{j}\right)\\ i,j=1,2,\cdots ,N. \end{array}$$

Assuming $A=\Omega +\gamma^{-1}I$, matrix *A* ought to be symmetric positive-definite according to Mercer’s Theorem. Then, parameter *a* and *b* can be solved via Equation (6):

(7) $$\hat{b}=\frac{\Theta^{T}A^{-1}Y}{\Theta^{T}A^{-1}\Theta },\;\hat{a}=A^{-1}\left(Y-b\Theta \right).$$

After substituting Equations (4) and (7) into Equation (1), the regression model of LSSVM is obtained:

(8) $$f\left(x\right)=\sum_{i=1}^{N}{\hat{a}}_{i}\kappa \left(x,x_{i}\right)+\hat{b}.$$

Theoretically, any function which satisfies the Mercer condition can be a kernel function. The linear, polynomial, and radial basis function (RBF) are three common types of kernel function. In this paper, the RBF kernel,

(9) $$\kappa_{RBF}\left(x_{i},x_{j}\right)=\exp\left(-\frac{{∥x_{i}-x_{j}∥}^{2}}{2\sigma^{2}}\right),$$

is selected, where the only parameter $\sigma^{2}$ is the width of kernel.

### 2.2. The Hybrid Whale Optimization Algorithm (hWOA)

#### 2.2.1. Whale Optimization Algorithm

The selection of two parameters γ and $\sigma^{2}$ makes a difference to the performance of LSSVM. Optimization algorithms are practically utilized to search optimal parameters by scholars to avoid trail blindness and obtain better performance of the model [10,21,30].

Meta-heuristic algorithms have been applied for optimization in many fields, such as mathematics, data science, and engineering. The reason why meta-heuristic algorithms become increasingly attractive can be attributed to three characteristics: simplicity, flexibility, and the ability to avoid local optima. The WOA is a swarm-based meta-heuristic optimization algorithm which undoubtedly inherits the advantages of meta-heuristic algorithms. Inspired by the humpback whale, Mirjalili and Lewis proposed the WOA by imitating the hunting pattern called *bubble-net preying*, which is only practiced by humpback whales. The preying behavior of humpback whales can be categorized into two main processes: searching for prey and the bubble-net attacking method. The two maneuvers just correspond to the exploration phase and exploitation phase, respectively, in meta-heuristic algorithms.

In the first maneuver, humpback whales will randomly select a reference whale and then move away by encircling it. The WOA represents this behavior by the following equations:

(10) $$\vec{D}=\left|\vec{C}\cdot {\vec{WX_{t}}}^{*}-\vec{WX_{t}}\right|$$

(11) $${\vec{WX}}_{t+1}={\vec{WX}}_{t}^{*}-\vec{A}\cdot \vec{D}$$

where *t* indicates the current iteration, $\vec{WX}$ is the position vector, and ${\vec{WX}}^{*}$ is the optimal position obtained so far. $\vec{A}\;,\;\vec{C}$ are both coefficients, and they can be calculated as follows:

(12) $$\vec{A}=2\vec{a}\cdot \vec{r_{1}}-\vec{a}$$

(13) $$\vec{C}=2\cdot \vec{r_{2}}$$

where $\vec{a}$ is linearly decreases along with the iteration between [2, 0], and $\vec{r_{1}}$, $\vec{r_{2}}$ are random vectors in range of [0, 1].

Humpback whales will perform the following maneuver (the bubble-net attacking method) after obtaining the approximate location of prey. In this maneuver, humpback whales will approach toward the prey using two different movement patterns: a shrinking circle and a spiral-shaped path. The model of the former path scheme in the WOA is nearly the same as Equations (10)–(13), except random vector $\vec{A}$ in Equation (11) is in the range [−1, 1] to force search agents to move toward the targets. For the latter scheme, the WOA mimics the spiral-shaped path by creating a spiral equation between the target and search agent as follows:

(14) $${\vec{WX}}_{t+1}=\vec{D^{{}^{\prime }}}\cdot e^{bl}\cdot \cos\left(2\pi l\right)+{\vec{WX}}_{t}^{*},$$

(15) $$\vec{D^{{}^{\prime }}}=\;\left|{\vec{WX}}_{t}^{*}-{\vec{WX}}_{t}\right|,$$

where

$\vec{D^{{}^{\prime }}}$ indicates the distance between the search agent and the target (the optimal position obtained so far),
*b* is a random constant for defining the shape of spiral,
*l* is a random number in [−1, 1].

Humpback whales will take two path schemes simultaneously when approaching the prey; thus, the WOA assumes the possibility of each scheme happening is 50% during the optimization. The mathematical model of the bubble-net attacking method is as follows:

(16) $${\vec{WX}}_{t+1}=\left\{\begin{array}{cc} \; & {\vec{WX}}_{t}^{*}-\vec{A}\cdot \vec{D}\;\;\;\;\;if\;p<0.5\\ \; & \vec{D^{{}^{\prime }}}\cdot e^{bl}\cdot \cos\left(2\pi l\right)+{\vec{WX}}_{t}^{*}\;\;\;if\;p\geq 0.5 \end{array}\right.$$

where *p* is a random number in [0, 1].

As shown above, the fitness is the only factor by which to judge a position as better or not. Therefore, cost functions are supposed to reflect the differences between output and real values. Generally, $L_{1}$ loss (mean absolute error, MAE):

$$\mathrm{MAE}\left(f,D\right)=\frac{1}{m}\sum_{i=1}^{m}\;|f\left(x_{i}\right)-y_{i}|$$

and $L_{2}$ loss (mean square error, MSE):

$$\mathrm{MSE}\left(f,D\right)=\frac{1}{m}\sum_{i=1}^{m}{\left(f\left(x_{i}\right)-y_{i}\right)}^{2}$$

are selected to calculate the fitness on regression tasks. Outliers cause greater loss to MSE than MAE; therefore, the model will try to fit outliers to reduce the cost [31]. The F/T sensors focus on static force, and fewer outliers will make a subtle influence on measurements. Thus, MAE is selected as the cost function in hWOA.

Furthermore, the cross-validation strategy is adopted to eliminate accidental error while calculating the fitness of the algorithm. Cross-validation randomly divides data into *k* (10 by default) disjoint sets, and then the *i*th set is used to estimate the performance of the model trained on other k−1 sets. Finally, the costs of all *k* sets are combined by average to obtain the fitness of the model.

#### 2.2.2. The Simulated Annealing Algorithm (SA)

Annealing is a process which toughens steel or glass by gradually heating and cooling. The SA algorithm combines annealing and the metropolis sampling criterion to obtain a strong ability to escape from local optima. The SA tries to make particles travel around all positions in solution space by setting a high initial temperature. Then, the temperature will gradually decrease to reduce the activeness of particles until the global optimal solution is obtained.

The standard SA is performed in following three steps:

##### Initialization

Set initial values: $x^{*}=x_{0}$, $T=T_{\max}$, and *k*, where

$x^{*}$ is the position of search agent in the *i*th iteration;
*T* and $T_{\max}$ indicate the current temperature and initial maximum temperature, respectively;
*k* is a constant in [0, 1] to control the decreasing speed of temperature.

##### Annealing

Select a point $x_{i}$ in solution space, and then compare its fitness $f(x_{i})$ with $f(x^{*})$, where f(·) is the cost function. If $f(x_{i})$ is no more than $f(x^{*})$, then $x^{*}=x_{i}$; otherwise, if $f(x_{i})$ is greater than $f(x^{*})$, the SA will accept $x_{i}$ to be the current optimal solution with the possibility of $\exp(-\left(f\left(x_{i}\right)-f\left(x^{*}\right)\right)/k\cdot T)$.

##### Termination

The SA turns back to step (2) repeatedly until the maximum iteration or target temperature is satisfied.

#### 2.2.3. Hybridization

The standard WOA takes a greedy strategy in the exploitation phase. If $fitness({\vec{WX}}_{t})$ is smaller than $fitness({\vec{WX}}_{t}^{*})$, ${\vec{WX}}_{t}^{}$ will become the best position; otherwise, if $fitness({\vec{WX}}_{t})$ is greater than $fitness({\vec{WX}}_{t}^{*})$, ${\vec{WX}}_{t}^{}$ will just be ignored. The hWOA performs the same as standard WOA in the former condition, but updates the best position according to randomness and the distance between the current position and the best one in the latter condition. To be specific, if $fitness({\vec{WX}}_{t}^{})$ is smaller than $fitness({\vec{WX}}_{t}^{*})$, hWOA will update the best position with ${\vec{WX}}_{t}^{}$:

(17) $$\begin{array}{cc} {\vec{WX}}_{t}^{*} & ={\vec{WX}}_{t}^{},\\ fitness({\vec{WX}}_{t}^{}) & \leq fitness({\vec{WX}}_{t}^{*})\;. \end{array}$$

when $fitness({\vec{WX}}_{t})$ is greater than $fitness({\vec{WX}}_{t}^{*})$, ${\vec{WX}}_{t}^{}$ could become the best position in the possibility of $P_{trans}$, which is the transfer possibility and defined as follows:

(18) $$P_{trans}=\exp\left(-\frac{fitness\left({\vec{WX}}_{t}^{}\right)-fitness\left({\vec{WX}}_{t}^{*}\right)}{T}\right)\;$$

where *T* indicates the system temperature. Equation (18) demonstrates that the transfer possibility $P_{trans}$ is inversely proportional to the distance between ${\vec{WX}}_{t}^{*}$ and ${\vec{WX}}_{t}^{}$, and directly proportional to the system temperature *T*.

In the progress of optimization, the search agents will get closer to the optima with more iterations, and $P_{trans}$ will increase as well. The hWOA will set a high initial temperature $T_{0}$ at the beginning to keep a balance between stability and exploring ability, and then exponentially decrease *T* along with iterations as follows:

(19) $$T_{t+1}=k\cdot T_{t}\;,$$

where $T_{t}$ is the system temperature of the *i*th iteration; k∈[0,1] is a coefficient to control the decreasing rate of *T*. The pseudo-code of hWOA is presented in Algorithm 1.
**Algorithm 1:** Hybrid Whale Optimization Algorithm**Inputs:**Initialize the population of whales $X_{i}\;\left(i=1,2,\ldots ,n\right)$,
Initialize the parameters $\alpha ,p,l,\vec{A},\vec{C}$ and $X_{curr}^{*}$**Outputs:**The optimal position $X^{*}$**Process:**
1.Calculate fitness of all search agents2.**while** (*t* < maximum number of iterations) || (stop condition):3.    Update *p* and flag4.    **for** each search agent:5.      **if** p>0.5:6.           **if** $\left|A\right|>1$:7.                Update the position of the current search agent by Equation (11)8.           **else if** $\left|A\right|\leq 1$:9.                Select a random search agent ${\vec{X}}_{rand}$10.                Update the position of the current search agent by Equation (15)11.           **end**12.      **else if** p≤0.5:13.           Update the position of the current search agent by Equation (14)14.      **end if**15.      **if** $fitness\left({\vec{X}}_{curr}\right)<fitness\left({\vec{X}}_{curr}^{*}\right)$: ${\vec{X}}_{curr}^{*}={\vec{X}}_{curr}$16.      **else if** $fitness\left({\vec{X}}_{curr}\right)\geq fitness\left({\vec{X}}_{curr}^{*}\right)$:17.           **if** $flag>P_{trans}$: **do nothing**18.           **else if** $flag\leq P_{trans}$: ${\vec{X}}_{curr}^{*}={\vec{X}}_{curr}$19.           **end if**20.      **end if**21.    **end for**22.    Check if any search agent travels beyond the search space and amend it23.    t=t+124.    T=k·T *% Update the system temperature by Equation (18)*25.**end while**26.**return**${\vec{X}}^{*}$

### 2.3. Improved Trimmed Bagging

Trimmed bagging is a modified version of the bagging algorithm. Since being proposed by Breiman in 1996, the bagging algorithm took a place in ensemble learning field due to its effectiveness and flexibility. The bagging algorithm can outperform a single learner under the limitation that the base learner should be both good on average and unstable to the disturbances of training datasets [32]. Therefore, decision trees, a typically unstable base learner, are proven to be well compatible with the bagging algorithm [33]. On the other hand, if stable algorithms, such as SVM and logistic regression, are selected to be base learners, the performance of bagging could be equivalent or even worse than that of the base learner [34]. To extend the compatibility of bagging to stable algorithms, Joossens and Croux improved the standard bagging algorithm with an intuitive idea: the trimmed bagging only averaging over those “good” base learners instead of all base learners. The complete process of trimmed bagging can be presented as follows:

1. The algorithm begins by applying bootstrap sampling on the given dataset. Assuming $D=\left\{\left(x_{1},y_{1}\right),\left(x_{2},y_{2}\right),\ldots ,\left(x_{m},y_{m}\right)\right\}$ denotes the original training datasets, we can obtain the bootstrap distributions $D_{i},\;i=1,2,\ldots ,T$, where *T* indicates the number of base learners.
2. Train all base learners with the corresponding subsets:
  (20) $$h_{i}=L\left(D,D_{i}\right),\;i=1,2,\cdots ,T$$
  where $L(D,D_{i})$ stands for training the base learning algorithm L with dataset *D* under distribution $D_{i}$, $h_{i}$ is the *i*th trained learner, and *T* is the number of base learners.
3. Calculate the quoted error (QE) of each base learner using OOB sampling:
  (21) $$\begin{array}{cc} QE_{\left(i\right)}= & QE(L\left(D,D_{i}\right))\\ = & \underset{\left(x_{k},y_{k}\right)\in \left(D,D_{i}\right)}{average}\left|\frac{f\left(x_{k}\right)-y_{k}}{y_{FS}}\right|, \end{array}$$
4. Sort all base learners ascendingly according to their QEs:
  $$\begin{array}{cc} QE(L\left(D,D_{1}^{oob}\right)) & \leq QE(L\left(D,D_{2}^{oob}\right))\\ & \leq \cdots \leq QE(L\left(D,D_{j}^{oob}\right))\;, \end{array}$$
  where j=1,2,⋯,T indicates the quoted error of $D_{j}^{oob}$ at the *j*th iteration for all base learners.
5. Trim off the “worst” learners at the portion of α; then average them over the remainder to obtain the ensemble learner:
  (22) $$h_{trim}=\underset{1\leq j\leq \left\lfloor (1-\alpha )T\right\rfloor \;\;\;}{\;averageL(D,D_{b})}\;\;$$

As shown above, the performance of the trimmed bagging is directly influenced by the trim portion α: too large an α will cause too few base learners to average over, and the bagging method is meaningless. On the other hand, too small an α will increase the risk of involving some “worst” learners. In standard trimmed bagging, the trimmed portion is fixed and less than 0.5. Obviously, the portions of the “worst” learners in ensemble models are various; a fixed trim portion α cannot be suitable for all situations. An adaptive strategy is designed to choose the trim portion α to improve the robustness of the model.

The adaptive trimming strategy chooses the portion α by minimizing the variance of QEs of all base learners involved in averaging. To be specific, it calculates the mean and variance, respectively, of different trim counts by using the following formulas:

(23) $$Mean\left(k\right)=\frac{1}{n-k}\sum_{i=1}^{n-k}QE_{\left(i\right)},$$

(24) $$Var\left(k\right)=\frac{1}{n-k}\sum_{i=1}^{n-k}{\left[QE_{\left(i\right)}-Mean\left(k\right)\right]}^{2},$$

where *k* indicates the number of trimmed base learners, and $QE_{\left(i\right)}$ stands for the quoted error of the *i*th base learner ordered by QE. Then, choose the optimal trim count $k^{*}$, which minimizes the variance:

(25) $$k^{*}=\left\{\;k|\min Var\left(k\right)\;,\;\left\lceil n/2\right\rceil \leq k\leq n\;\right\}.$$

Correspondingly, the optimal trim portion $\alpha^{*}$ can be obtained as follows:

(26) $$\alpha^{*}=\frac{k^{*}}{n}$$

After trimming off the base learners at portion $\alpha^{*}$, the standard trimmed bagging will apply arithmetic averaging over the remaining base learners. The arithmetic average distributes each base learner with the same weight, and learners contribute equally to the model. However, base learners perform diversely, and we think the better a base learner performs, the greater its weight should be. Based on this idea, the weights of base learners are equivalent to the inverses of their QEs:

(27) $$w_{i}=\frac{1/QE_{i}}{\sum_{j=1}^{N-k^{*}}\left(1/QE_{j}\right)}\;,$$

where $w_{i}$ indicates the weight of the *i*th base learner after trimming.

After obtaining the optimal trim portion α and the weight of each base learner, the improved trimmed bagging assembles the outputs of base learners as follows:

(28) $$h_{trim}=\frac{\sum_{j=1}^{\left\lceil (\;1-\alpha^{*})\cdot N\right\rceil }w_{i}\cdot L\left(D,D_{j}\right)}{\sum_{k=1}^{\left\lceil (\;1-\alpha^{*})\cdot N\right\rceil }w_{k}}$$

The pseudo-code of the improved trimmed bagging algorithm can be presented as Algorithm 2.
**Algorithm 2:** Improved Trimmed Bagging Algorithm**Inputs:**Dataset $D=\left\{\left(x_{1},y_{1}\right),\left(x_{2},y_{2}\right),\ldots ,\left(x_{m},y_{m}\right)\right\}$,
Base learning algorithm L,
The trimming portion α,
Number of base learners *T***Outputs:**The ensemble learner $h_{trim}$**Process:**
1.Initial the distributions $\;D_{i}\;\left(i=1,2,...,T\right)$ by applying bootstrap sampling over dataset *D*2.Initial out-of-bag samples $\;D_{i}^{oob}\;\left(i=1,2,...,T\right)$ correspond to distribution $\;D_{i}$3.**for** i=1,2,⋯,T4. $h_{i}=L\left(D,D_{i}\right)$
*%Train the base learning algorithm by distribution*
$D_{i}$
5. $qe_{i}=QE\left(L\left(D,D_{i}^{oob}\right)\right)$
*%Calculate quoted error of current learner*
6.**end**7.Sort all learners ascendingly according to quoted error.8.Find the optimal trim portion $\alpha^{*}$ by Equation (23)–(26).9.Calculate the weight $w_{i}$ of each base learner by Equation (27).10.$h_{trim}=\left[\sum_{j=1}^{\left\lceil (\;1-\alpha^{*})\cdot N\right\rceil }\;w_{i}\cdot L\left(D,D_{j}\right)\right]/\left[\sum_{k=1}^{\left\lceil (\;1-\alpha^{*})\cdot N\right\rceil }\;w_{k}\right]$*%Average on the remaining base learners*11.**return**$h_{trim}$

## 3. Hybrid WOA-LSSVM Ensembled by Improved Trimmed Bagging

This section introduces how the presented algorithms interconnect and how to constitute the proposed temperature compensation model.

1. We prepared the datasets with a high–low temperature experiment. The input vector was formed by a series of ambient temperatures (*T*), and the voltage signal (*V*) of the strain gage sensor constituted the output vector. Then, 80% of origin data were randomly separated for training and the remaining 20% were prepared for testing. More details about the datasets are shown in the High–Low Temperature Experiment section.
2. We divided the training set into *N* distributions by bootstrap sampling and obtained base learners by training LSSVM on each distribution. Two parameters of LSSVM, the regulation item (γ) and the width of RBF kernel ($\sigma^{2}$), were tuned by hWOA in this process.
3. We calculated quoted error (QE) of each base learner according Equation (21) by out-of-bag (OOB) sampling; then, we determined the optimal trim portion ($\alpha^{*}$) and weight ($w_{i}$) by the adaptive trim strategy.
4. We assembled the final model by trimming off and averaging over the outputs of base learners according to $\alpha^{*}$ and $w_{i}$.

**Figure 1:**
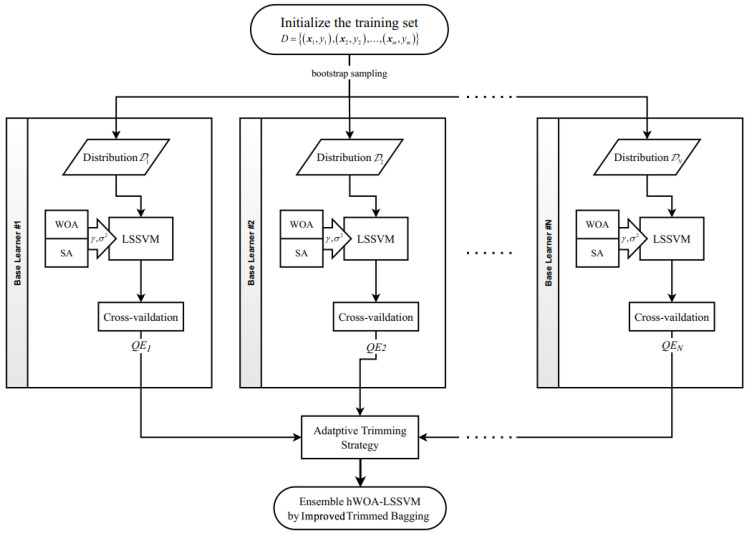
Flowchart of ensemble hWOA-LSSVM based on improved trimmed bagging.

The flowchart of the hybrid whale optimization algorithm optimized least-squares support vector machine (hWOA-LSSVM) ensembled by improved trimmed bagging is demonstrated in Figure 1.

## 4. Experiment

All experiments were conducted on a six-axis force/torque sensor designed by the Institute of Intelligent Machines (IIM), Chinese Academy of Sciences (CAS). The F/T sensor consists of strain gauges and a novel double E-shape elastic body. The rated ranges of the F/T sensor are Fx=Fy=±600 N, Fz=±1000 N, and Mx=My=Mz=±30 N·m.

### 4.1. Calibration and Decoupling

A calibration experiment was conducted to obtain the transfer expression from outer stimuli to voltage signal responded bthe y sensor. Assuming $U={\left[u_{1},u_{2},\ldots ,u_{6}\right]}^{T}$ indicates the output vector, which consists of the voltage of each dimension, and $L={\left[Fx,Fy,Fz,Mx,My,Mz\right]}^{T}$ indicates the measured load vector, the transfer expression can be presented by the following equation:

(29) L=W·U+B.

where *W* is the weight matrix, which is also called the calibration matrix, and *B* is the bias vector.

The basic procedures for multi-axis F/T sensor calibration apply a series of known loads which increase from minimum to maximum rated range with a certain step. The voltage responded by the sensor is recorded at each sample point. The above procedures were repeated for three times in this calibration experiment, and all record data were used for calibration and decoupling. Configurations of calibration experiment are shown in Table 1, and the environment temperature and humidity are 25 °C and 60%, respectively.

After all calibration procedures completed, the least-squares (LS) algorithm was adopted to calculate the calibration matrix *W* and bias vector *B*, which are as follows:

(30) $$W=\left(\begin{array}{cccccc} 643.017 & -7.270 & 148.091 & -0.068 & -430.623 & 6.890\\ 544.952 & 46.697 & -170.297 & 28.248 & -349.738 & -452.637\\ -11.149 & 10.105 & 266.068 & 2.265 & 11.459 & -5.324\\ 6.419 & -6.059 & -2.374 & 0.359 & -4.202 & -5.568\\ -1.132 & 0.109 & -2.443 & 0.010 & 5.178 & 0.060\\ 0.374 & -0.023 & -4.341 & -0.017 & -0.111 & 6.426 \end{array}\right),$$

(31) $$B={\left[-258.561,-202.996,-559.61,-2.9339,0.3304,-2.7932\right]}^{T}.$$

Finally, the transfer expression could be obtained by substituting *W* and *B* into Equation (29).

### 4.2. High–Low Temperature Experiment

A high–low temperature experiment was conducted to analyze the measurement error of sensors caused by temperature drift and obtain the data for the training model. Wang shows that exerting loads on F/T sensors makes no difference to the temperature drift phenomenon [35]; therefore, the sensors were not loaded with any force/toque during the temperature experiment.

**Figure 2:**
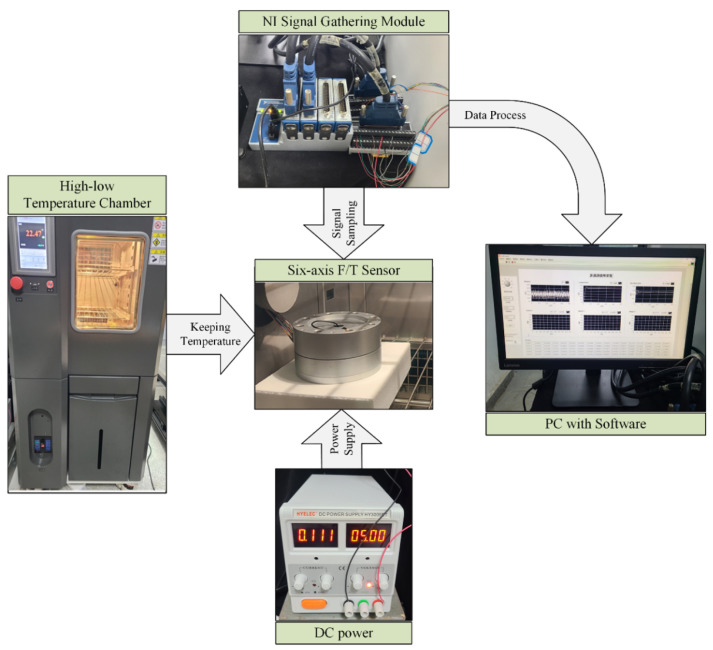
Temperature experiment configuration.

The six-axis F/T sensor was placed in a high–low temperature chamber and run at 5V DC voltage in the experiment. The temperature in the chamber was varied from −30 to 70 °C, and kept for 2 h at thirteen temperature sampling points (marked as Ts): −30, −20, −10, 0, 10, 20, 25, 30, 35, 40, 50, 60, and 70 °C. The gathering module sampled about 450 outputs (marked as $U_{o}$) of the F/T sensor during each Ts and transmitted them to the PC for processing and storage. The temperature experiment configuration is demonstrated in Figure 2.

The sensor measurement error $E_{m}$ is convenient for comparing, which can be defined as follows:

(32) $$E_{m}=\frac{\theta_{T}-\theta_{ref}}{\theta_{FS}}\times 100\%$$

where

$\theta_{T}$ is the measured value of the F/T sensor under *T* °C;
$\theta_{ref}$ denotes the measured value under the temperature of calibration, which means 25 °C here;
$\theta_{FS}$ denotes the full scale of the corresponding dimension.

**Figure 3:**
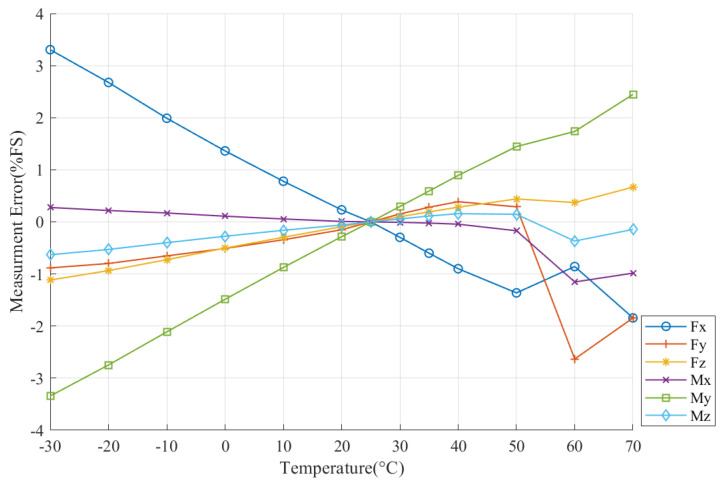
Measurement error before compensating by EhW-LSSVM.

Measurement errors caused by temperature in all six dimensions before compensation are illustrated in Figure 3.

As is shown in Figure 3, all dimensions of the F/T sensor suffer characteristic drift, which consists of both linear and nonlinear components. The relation between $E_{m}$ and $T_{s}$ presents more nonlinear features when ambient temperature is higher than 50 °C. In addition, Fy, Fz, My, and Mz have positive correlations with $E_{m}$ and $T_{s}$; and Fx and Mx have negative correlations with them.

Overall, Fz, Mx, and Mz have less $E_{m}$ and lower linearity than Fx, Fy, and My. Mx had the lowest $E_{m}$, which is no more than $0.6{\%}_{FS}$, and Fx had the largest $E_{m}$, which reached $3.3{\%}_{FS}$ at −30 °C. The measurement error of the F/T sensor caused by temperature variation was manifest; therefore, temperature compensation is vital for six-axis F/T sensors to meet the requirements of space manipulator control.

### 4.3. F/T Sensor Temperature Compensation

#### 4.3.1. Model Training

The original dataset gathered in the temperature experiment was divided into 80% and 20% for training and testing, respectively. To evaluate the proposed model better, several algorithms, including standard support vector regression (Std-SVR), LSSVM optimized by particle swarm optimization (PSO-LSSVM), LSSVM optimized by the standard whale optimization algorithm (WOA-LSSVM), and the RBF neural network optimized by the standard whale optimization algorithm (WOA-RBFNN) were compared.

In the RBFNN, the number of neurons in the hidden layer was 13, and the centers of RBF function were set to −30,−20,−10,0,10,20,25,30,35,40,50,60, and 70, which correspond to the 13 sampling points in the aforesaid high–low temperature experiment.

The parameters for all algorithms were as identical as possible; all the parameters are demonstrated in Table 2. Additionally, the mean square error (MSE) was used as the fitness function to evaluate the performances of algorithms.

#### 4.3.2. Compensation

**Figure 4:**
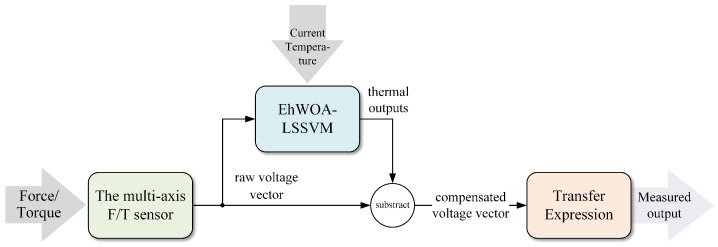
Compensating procedures of ensemble hWOA-LSSVM based on improved trimmed bagging.

We compensated for the characteristic drift by connecting the trained temperature compensation model to outputs of the F/T sensor. To be specific, as is shown in Figure 4, the multi-axis F/T sensor changed force and torque into voltage outputs, and then the compensation model calculated thermal outputs (which could be either positive or negative) according to the voltage outputs and current temperature. Compensation outputs can be obtained by subtracting thermal outputs from raw outputs of the sensor. Finally, the measured values were obtained by substituting the compensation outputs into the transfer Expression (29).

### 4.4. Compensation Results and Analysis

The compensation results of various dimensions on the training set and testing set are shown in Table 3 and Table 4, respectively. As demonstrated in Table 3, in the training set, PSO-LSSVM performed best in four dimensions (Fx, Fy, Fz, Mx), and EaW-LSSVM had the worst fitness in the other two dimensions (My, Mz). The ensemble model EhW-LSSVM performed slightly better than the single model WOA-LSSVM in the training set. The performance of WOA-RBFNN was average overall, and Std-SVM was inferior to other algorithms.

In the testing set, as Table 4 shows, the EhW-LSSVM gained the lowest fitness in four dimensions (Fy, Fz, Mx, Mz), and the PSO-LSSVM performed better in Fx and My. The performance of the EhW-LSSVM was better than that of the single learner WOA-LSSVM in most dimensions. In addition, the fitness of all dimensions except Mz obtained by the WOA-RBFNN deteriorated greatly in the testing set, which indicates that the RBFNN suffers severe over-fitting under the same parameter configuration as the other algorithms.

We can draw the following inferences by analyzing the temperature compensation results: first, the EhW-LSSVM shows competitive predictive ability because of its reliable performance on the testing set and training set. Second, using the bagging ensemble method, the ensembled model did not show worse performance in training than the single learner, but obtained better predicting ability.

**Figure 5:**
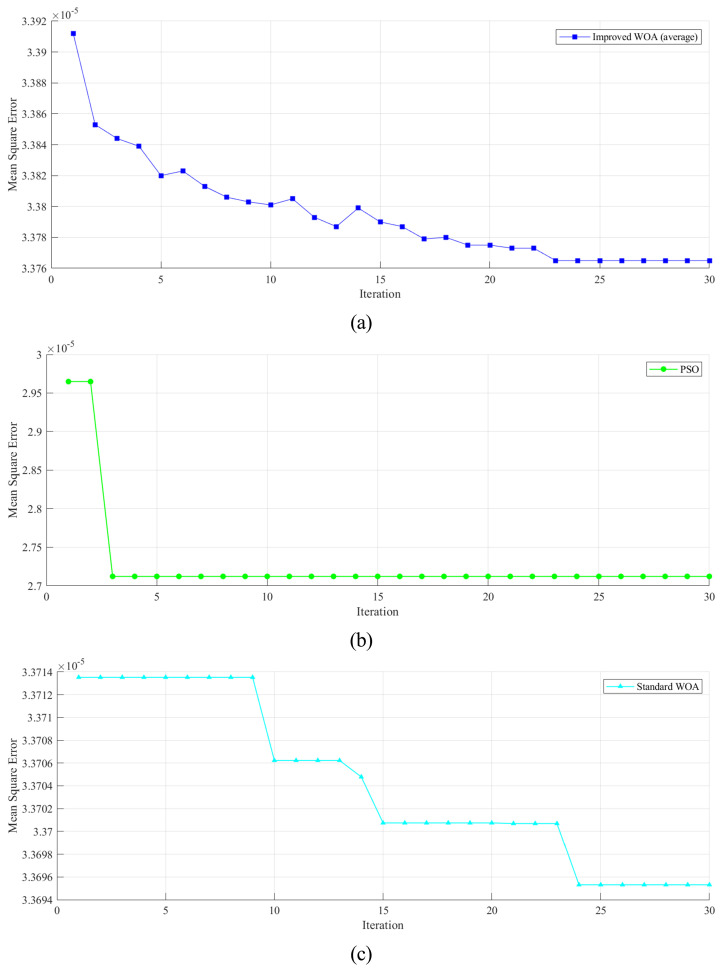
Best fitness obtained on the training set by each algorithm. (**a**) Best fitness obtained throughout iterations by improved WOA. (**b**) Best fitness obtained throughout iterations by PSO. (**c**) Best fitness obtained throughout iterations by standard WOA.

The best fitness of Fz in each iteration obtained on the training set was taken as an example to evaluate the search convergence characteristics. Additionally, the convergence curves of EhW-LSSVM (average on all base learners), PSO-LSSVM, and WOA-LSSVM are demonstrated in Figure 5. As depicted in Figure 5a,c, the hybrid whale optimization algorithm jumps out of local optima but drops in fitness sometimes. It is inferred that, benefiting from the hybridization of the SA, the hWOA has a remarkable ability to jump out of local optima and still retains good global searching ability. In addition, the convergence characteristic of PSO in Figure 5b shows that the PSO converges too early and has a poor ability to get out of local optima.

**Figure 6:**
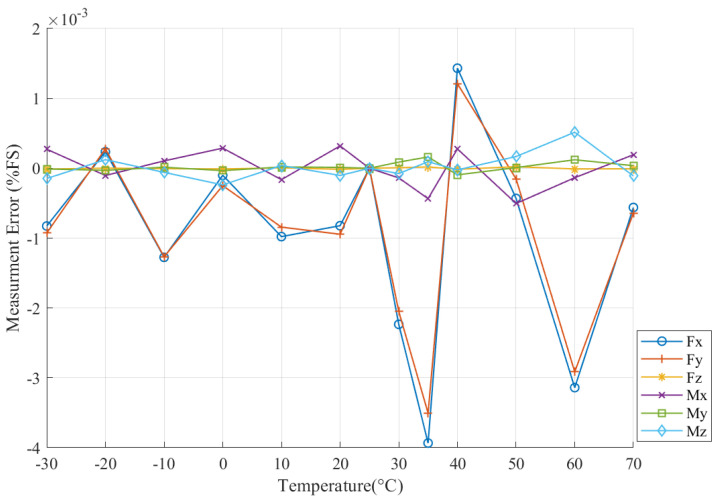
Measurement errors after compensating by EhW-LSSVM.

After compensating with the EhW-LSSVM, the measurement errors caused by temperature variation are demonstrated in Figure 6. Overall, the measurement errors of all dimensions decreased dramatically after compensation by the EhW-LSSVM. Fz and My had the best compensating performances, whose maximum measurement errors were less than $2.2\times 10^{-5}{\%}_{FS}$ and $1.7\times 10^{-4}{\%}_{FS}$, respectively. The performances of Fx and Fy were somewhat inferior to those of other dimensions, and the maximum absolute measurement error of Fx was still less than $3.9\times 10^{-3}{\%}_{FS}$. In addition, the measurement errors of all dimensions showed no noticeable changes with temperature variation. Above all, the six-axis F/T sensor suffered from the temperature drift negligibly and met the request of cosmic operation after compensation by the EhW-LSSVM.

## 5. Conclusions

Our novel temperature compensating method consisting of LSSVM optimized by hybrid WOA and improved trimmed bagging was presented in this work for eliminating the characteristic drift of six-axis force/torque sensors in cosmic space. In addition, simulated annealing (SA) is applied to WOA to cover the shortage in exploiting. Furthermore, the optimal trim portion of trimmed bagging is determined by an adaptive trimming strategy, which automatically adjusts the trim portion according to the performances of base learners. Cross-validation and inverse quoted error are utilized to evaluate the model more accurately.

A high–low temperature experiment was conducted to investigate the impacts of temperature variation on six-axis F/T sensors and provide data for model training. The compensating results indicate that EhW-LSSVM possesses excellent predicting ability and dramatically decreased the measurement errors of six-axis F/T sensors to a level of $10^{-3}{\%}_{FS}$. The hybrid WOA showed better ability than standard WOA during the process with search optimal parameters. In addition, the adaptive trimmed bagging lifted the effect of a single model in the testing set while losing no accuracy in the training set. According to temperature compensating results and comparisons with other algorithms, the EhW-LSSVM algorithm is a feasible and competitive temperature compensating method for six-axis F/T sensors.

The compensating performance of the EhW-LSSVM is satisfactory, but the complexity of its structure is also high. In future research, we will aim to reduce the model’s complexity and try to integrate the presented EhW-LSSVM into compact six-axis F/T sensors.

## Figures and Tables

**Table 1:** Calibration experiment configuration.

Dimensions	Load Points	Units
Fx	−600, −400, −200, 0, 200, 400, 600	N
Fy	−600, −400, −200, 0, 200, 400, 600	N
Fz	0, 200, 600, 800, 1000	N
Mx	−30, −20, −10, 0, 10, 20, 30	N·m
My	−30, −20, −10, 0, 10, 20, 30	N·m
Mz	−30, −20, −10, 0, 10, 20, 30	N·m

**Table 2:** Parameters of all models.

Parameters	Std-SVR	EhW-LSSVM	PSO-LSSVM	WOA-LSSVM	WOA-RBFNN
γ	-	[0.01, 300]	[0.01, 300]	-	[0.01, 300]
$\sigma^{2}$	50	[1, 1000]	[1, 1000]	[1, 1000]	[1, 1000]
Maximum iteration	-	30	30	30	30
Count of search agents	-	20	20	20	20
Count of base learners	-	10	-	-	-

**Table 3:** Compensation results on the training set by different methods.

Parameters	Std-SVR	EhW-LSSVM	PSO-LSSVM	WOA-LSSVM	WOA-RBFNN
Fx	$2.6340\times 10^{-5}$	$1.4106\times 10^{-5}$	$1.0572\times 10^{-5}$	$1.4136\times 10^{-5}$	$1.2504\times 10^{-5}$
Fy	$9.7145\times 10^{-6}$	$9.0614\times 10^{-6}$	$6.1726\times 10^{-6}$	$9.0729\times 10^{-6}$	$6.8415\times 10^{-6}$
Fz	$4.4808\times 10^{-5}$	$3.3610\times 10^{-5}$	$2.7122\times 10^{-5}$	$3.3695\times 10^{-5}$	$2.9582\times 10^{-5}$
Mx	$4.6609\times 10^{-5}$	$4.5047\times 10^{-5}$	$3.2643\times 10^{-5}$	$4.5115\times 10^{-5}$	$3.5834\times 10^{-5}$
My	$3.2747\times 10^{-5}$	$1.0729\times 10^{-5}$	$1.6889\times 10^{-5}$	$1.0775\times 10^{-5}$	$1.8464\times 10^{-5}$
Mz	$6.4732\times 10^{-5}$	$2.9451\times 10^{-5}$	$3.1305\times 10^{-5}$	$2.9531\times 10^{-5}$	$3.5159\times 10^{-5}$

**Table 4:** Compensation results on the testing set by different methods.

Parameters	Std-SVR	EhW-LSSVM	PSO-LSSVM	WOA-LSSVM	WOA-RBFNN
Fx	$3.6100\times 10^{-5}$	$2.9417\times 10^{-5}$	$2.9311\times 10^{-5}$	$3.3722\times 10^{-5}$	$4.2461\times 10^{-2}$
Fy	$1.8291\times 10^{-5}$	$1.0058\times 10^{-5}$	$1.0456\times 10^{-5}$	$1.0266\times 10^{-5}$	$1.5098\times 10^{-3}$
Fz	$5.8494\times 10^{-5}$	$4.0726\times 10^{-5}$	$4.2809\times 10^{-5}$	$4.2876\times 10^{-5}$	$9.6410\times 10^{-2}$
Mx	$7.2215\times 10^{-5}$	$4.5470\times 10^{-5}$	$4.6506\times 10^{-5}$	$4.5767\times 10^{-5}$	$8.1106\times 10^{-2}$
My	$4.3461\times 10^{-5}$	$3.0991\times 10^{-5}$	$2.9197\times 10^{-5}$	$3.2692\times 10^{-5}$	$4.0547\times 10^{-2}$
Mz	$8.7759\times 10^{-5}$	$1.1178\times 10^{-5}$	$1.3900\times 10^{-5}$	$6.8330\times 10^{-5}$	$7.8865\times 10^{-5}$

## Data Availability

Not applicable.
